# Interplay Between Key Molecular Signaling Pathways and Immune Players Following Brain Irradiation

**DOI:** 10.1155/mi/8833271

**Published:** 2025-12-23

**Authors:** Seidu A. Richard

**Affiliations:** ^1^ Department of Biochemistry and Forensic Sciences, School of Chemical and Biochemical Sciences, C. K. Tedam University of Technology and Applied Sciences (CKT-UTAS), P.O. Box 24, Navrongo, Ghana, cktutas.edu.gh; ^2^ Institute of Neuroscience, Third Affiliated Hospital, Zhengzhou University, Zhengzhou, 450052, China, zdsfy.net

**Keywords:** immune, interplay, irradiation, radiotherapy, RIBI, signaling

## Abstract

Irradiation (IRT) has been used extensively for the diagnosis and treatment of primary as well as metastatic brain tumors. Although the susceptibility of the radiation‐induced brain injury or damage (RIBI) is partially associated with the sensitivity of particular cell types, pathways of injury in other types of brain disease or damage implicate the significance of the interaction between cell compartments. IRT‐induced double‐strand breaks (DSBs) are the utmost detrimental kind of DNA damage, which result in cell death as well as sustainable chromosomal reconfigurations following IRT exposure to the brain. It is worth noting that IRT‐induced DSBs, and stimulation of interplay of key signaling pathways such as mitogen‐activated protein kinases (MAPK), JAK/STAT, phosphoinositide‐3‐kinase/protein kinase B/AKT (PI3K‐PKB/AKT), protein 53 (p53), mammalian target of rapamycin (mTOR), NF‐kB, transforming growth factor beta (TGF‐*β*), tumor necrosis factor (TNF), as well as reactive oxygen species (ROS) to either trigger radiosensitization or radioresistance as well as RIBI mechanisms. Also, IRT is capable of influencing fundamental immune players like cluster of differentiation markers, the complement cascade, T cells, B cells, interleukins, as well as chemotactic cytokines. Thus, the aim of this review is to explicate the key molecular signaling and immune mechanisms associated with IRT‐induced neurological deficits following brain IRT.

## 1. Introduction

The efficacy of irradiation (IRT) for brain tumors is exceedingly dependent on the type as well as the magnitude of damage triggered by IRT on the tumor cells, as well as on their capability to repair radiation‐induced brain injury or damage (RIBI) [1–3]. The pathological mechanism is often referred to as the gliocyte hypothesis because all the brain gliocytes such as oligodendrocytes, astrocytes, and microglia are associated with the radioactive destruction process in distinctive ways, and RIBI is mostly white matter necrosis as well as demyelination [4, 5].

Furthermore, direct interaction between IRT and cellular components, such as DNA, as well as the generation of highly reactive species that disseminate damage inside and outside the IRT area through bystander effects are the consequences of cells being damaged by IRT [4]. Also, antioxidant, DNA damage repair, cell cycle arrest, as well as unfolded protein response are the mechanisms via which cells protect themselves against RIBI [6]. Although these cellular mechanisms offer protection to healthy tissue, the same mechanisms can have an undesirable influence on the efficacy of IRT for brain tumors.

Moreover, these mechanisms modify homeostatic molecular pathways as well as cell viability [7, 8]. The most critical component of IRT antitumor cell effects is the generation of reactive oxygen species (ROS) as well as nitrogen molecular species [8, 9]. Similarly, molecular endpoints for pathways such as tumor protein 53 (p53), transforming growth factor beta 1 (TGF‐*β*1), nuclear factor kappa B (NF‐*κ*B), phosphoinositide‐3‐kinase/protein kinase B/AKT (PI3K‐PKB/AKT), and B‐cell lymphoma 2 (Bcl‐2) involved in the cells’ coping with RIBI are often analyzed [9, 10].

Single IRT augmented several transcription factors such as cAMP response element‐binding protein (CREB), activator protein‐1 (AP‐1), and specificity protein 1 (Sp‐1) [11]. The underlying mechanisms of RIBI is a matter of debate. However, radiosensitivity of tissues to the depletion of one or more radiosensitive target cell populations has been implicated as a classic approach [12]. Also, blood–brain barrier (BBB) disruption and vascular damage suggest endothelial cells as targets, while demyelination implicates the significance of myelin‐producing oligodendrocytes [12].

Although the susceptibility of the RIBI is partially associated with the sensitivity of particular cell types, pathways of injury in other types of brain disease or damage implicate the significance of the interaction between cell compartments. Thus, this review elucidates the key molecular signaling and immune mechanisms associated with IRT‐induced neurological deficits following brain IRT.

The “boolean logic” was used to search for articles on the subject matter in PubMed, PMC, Web of Science, and Google Scholar, with strict inclusion criteria being the molecular signaling pathways and immune players associated with IRT‐induced neurological deficits or sequelae in the brain. Articles that discussed biomarkers, pathological mechanisms, as well as neuroprotection and therapeutic agents were excluded and discussed in separate papers. Strictly, only studies that described molecular signaling pathways and immune players associated with IRT‐induced neurological deficits or sequelae in the brain are discussed in this review paper.

## 2. DNA and IRT

IRT‐induced double‐strand breaks (DSBs) are the utmost detrimental kind of DNA damage, which result in cell death as well as sustainable chromosomal reconfigurations [13, 14]. Thus, cells have developed an efficient as well as quick DNA damage response (DDR) via DNA damage sensors (DDSs) to preserve genomic integrity [13, 14]. Furthermore, perfect DDSs are the first response proteins that are capable of identifying DNA damage as well as recruiting transducer proteins that provide signals to enzymes in response to the DNA damage [13, 15]. Also, DNA damage triggers a sequence of biochemical reactions in response to IRT damages as a signal and activates a multiplicity of cellular responses [16]. Therefore, DDSs as well as early signal transducers are very crucial in the detection of DNA damage [13, 17].

Specifically, IRT triggers chromosomal DSBs, which activate the DDR associated with the stimulation of DNA damage protein kinase ataxia‐telangiectasia mutated (ATM) and subsequently the generation of phosphorated H2A histone family member X (*γ*H2A.X), which function as a docking station, recruiting repair factors like p53 binding protein 1 (53BP1) to form IRT‐induced foci (IRIF), also referred to as DNA repair foci [16]. DNA damage repair deficiencies that result in assiduous DNA breaks are related to the stimulation of neuronal p53/p21 senescence pathways that trigger neurodegeneration [18].

It is worth noting that 53BP1 is extensively secreted with a homogenous/diffuse nuclear staining pattern, while *γ*H2A.X is only weakly secreted in noninjured neurons [14]. However, the nuclear dissemination of both *γ*H2A.X and 53BP1 becomes extremely intense, signifying the existence of *γ*H2A.X at DSB sites as well as the recruitment of 53BP1 to IRIF following IRT exposure [19]. Also, there is a quantitative correlation between *γ*H2A.X foci formation as well as DSBs after IRT exposure [19]. Specifically, *γ*H2A.X as well as 53BP1 foci are indicators of DNA breaks, and a decrease in the number of *γ*H2A.X as well as 53BP1 foci is a marker of DNA repair [20].

Notably, two molecules such as Rad50 and Rad54l2 are associated with DNA repair [14]. Functionally, Rad50 is fundamental for the action of DSB repair nuclease meiotic recombination 11 (MRE11), while Rad54l2, a member of the sucrose nonfermenting 2 (SNF2)‐like family of proteins associated with chromatin remodeling, DNA repair, as well as homologous recombination, is anticipated miR‐711 target [21]. IRT precipitously facilitated downregulation of neuronal Rad50 as well as Rad54l2, which correlated with upregulation of miR‐711 [14].

However, augmented concentrations of miR‐711, Rad50, as well as Rad54l2 was observed in the RNA‐induced silencing complex (RISC) [14]. Notably, the instruction of the miR‐711 blocker mitigated these changes, signifying that miR‐711 is a negative modulator of Rad50 as well as Rad54l2 [14]. Moreover, depletion of the deubiquitylating enzyme ubiquitin carboxyl‐terminal hydrolase isozyme L3 (UCHL3) leads to a decrease in chromatin‐binding as well as IRIF formation of Ku80 after DSB occurrence, relatively sensitizing tumor cells to IRT [22].

IRT triggered the accretion of autophagosomes as well as the radiosensitizing effect of autophagy‐related BECN1/BECLIN1 deficiency, leading to the disruption of nuclear translocation and Ku protein activity, resulting in the mitigation of DSB repair in malignant glioma [13]. BECN1 is an essential component of the PI3K‐3 complex, which influences membrane trafficking as well as restructuring associated with autophagy, endocytosis, phagocytosis, and cytokinesis [23].

Borealis (BORA), previously referred to as C13orf34, was phosphorylated by mediator of DNA damage checkpoint 1 (MDC1), resulting in annulment of IRT‐induced MDC1 foci formation, as well as downregulation of BORA augmented the resistance to IRT, possibly due to a quicker rate of DSB repair [24]. Typically, DNA damage triggered by IRT recruits MDC1 to sites of injury within ~1 min post‐IRT, providing a *γ*H2AX‐dependent interaction platform for recruiting other DNA damage repair proteins, like ATM and Nijmegen breakage syndrome 1 (NBS1), as well as the glycolytic enzyme 6‐phosphofructo‐2‐kinase/fructose‐2,6‐biphosphatase 3 [13].

Generally, *γ*H2AX correlates better with characteristics of a DDS than the secretion of DSB repair genes [25]. Presently, *γ*H2AX is extensively utilized as a marker to identify IRT‐induced DSB repair via immunofluorescent staining of foci or immunocytochemistry [19, 25]. Also, *γ*H2AX persisted after exposure to IRT in association with several radiosensitizing drugs, implying that this sensor could be utilized to monitor cancer treatment as well as to tailor cancer therapy [26]. Moreover, anti‐*γ*H2AX monoclonal antibodies and immunofluorescence hybridization techniques were capable of visualizing *γ*H2AX localization at sites of DNA damage [27].

It is worth noting that *γ*H2AX foci could be visualized accurately, but once the DNA damage was repaired, the foci were eliminated even with an extremely low dose of IRT exposure [28]. It was further observed that *γ*H2AX foci signify DSBs in a 1:1 ratio and can be utilized as a biomarker for DNA damage [29]. Also, the NBS1/hMRE11/hRad50 complex was very fundamental in DNA DSB sensing as well as the signal transduction triggered by X‐IRT [30]. Moreover, a persistent upsurge in IRT‐induced NBS1 foci formation was associated with an augmentation in the incidence of spontaneous chromosome aberrations [31].

Additionally, the NBS1/hMRE11/hRad50 complex interrelation with the DNA ligase III *α*/XRCC1 complex was also associated with the nonhomologous end‐joining (NHEJ) pathway in tumor cells following IRT [32]. Thus, the key DDS proteins are *γ*H2AX, 53BP1, NBS1, BRCA1/2, as well as Ku. The expression of DSB repair genes was expressively diminished, while the concentration of *γ*H2AX was augmented and sustained at an extreme concentration in diffuse intrinsic pontine glioma cells during the evaluation of the influence of histone demethylase blockade on genes associated with DSB repair [33]. Studies involving DIPG, *γ*H2AX, and DSB repair following cranial IRT in neonatal or juvenile models are needed.

Thus, these DDSs localize to the sites of DSBs within a few seconds or minutes after IRT exposure and establish microscopically visible nuclear domains known as IRIF [13]. Also, DDS proteins are capable of modifying the adjacent damage sites via techniques the phosphorylation of *γ*H2AX [13]. Furthermore, the DDS proteins are capable of modulating each other as well as recruiting other proteins to the injury site to form the NBS1/hMRE11/hRad50 protein complex [13]. Studies involving cranial IRT and childhood brain tumors and key DDS proteins such as *γ*H2AX, 53BP1, NBS1, and BRCA1/2, as well as Ku and DSB repair following IRT, are needed.

## 3. Mitogen‐Activated Protein Kinases (MAPK) Signaling

The MAPK family of kinases comprises extracellular signal‐regulated kinase (ERK1/2) or (p44 and p42), p38, and c‐Jun N‐terminal kinases (JNKs), also known as stress‐activated protein kinase (SAPK) [34]. MAPKs typically function at the level of the cytoplasm as well as nucleus by regulating transcriptional responses at the tissue level or cellular level once they are stimulated [35]. Thus, these pathways influence diverse extracellular stimuli as well as modulate cell cycle progression, proliferation, transcription, differentiation, cytokinesis, senescence, cell death, migration, GAP junction formation, actin as well as microtubule networks, neurite extension and cell adhesion motility, stress response, survival, as well as apoptosis [34].

Notably, each member of the MAPK superfamily kinases is associated with a distinct signal cascade though they are functionally interrelated in certain contexts [36]. The JNKs are primarily c‐Jun kinases targeting Ser63 as well as Ser73 in the c‐Jun N‐terminus, while the ERK1/2 as well as p38 are fundamental for c‐fos phosphorylation [37]. Interestingly, a cross‐talk between JNK and the ERK1/2 signaling pathways was observed when ERK1/2 was functionally switched for JNK1/JNK2 to facilitate phosphorylation of Ser63/73 on c‐Jun protein in vitro and in mouse fibroblasts [38].

**Figure 1:**
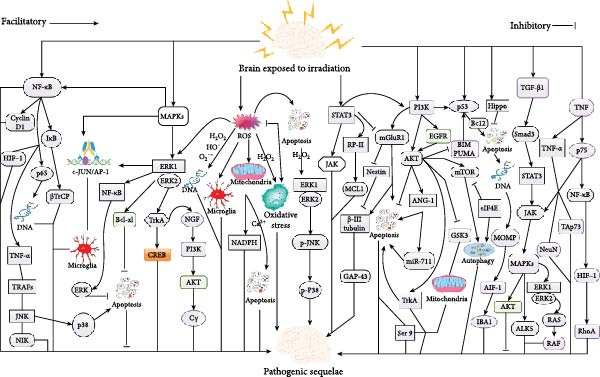
An illustration showing the key molecular signaling mechanisms associated with IRT‐induced neurological deficits or sequelae following brain IRT. *Note:* Refer to the text for detailed explanations. Also, refer to the general abbreviation list for the meaning of abbreviations.

The c‐JUN mostly constitutes homodimers or heterodimers with other AP‐1 proteins and binds to precise DNA components in promoters to modulate target genes [39]. Several AP‐1 proteins are modulatory targets of MAPKs, and stimulation of AP‐1 proteins is one of the cellular responses to IRT (Figure 1) [39]. Remarkably, the c‐JUN, an element of the AP‐1 transcription factors, modulates the secretion of numerous genes, comprising both inflammatory as well as cytokine genes, which contain consensus AP‐1 binding sites in their promoter regions (Figure 1) [36, 39]. Thus, c‐JUN is a critical modulator of neural cell death as well as neuroinflammation [40].

The ERK1/2 activation occurs at the cytosol, plasma membrane, as well as endo‐membranes, and its subcellular localization determines the ERK module sensitivity [34, 41, 42]. Also, IRT‐induced G2/M checkpoint response necessitates the stimulation of ERK1/2 signaling (Figure 1) [43]. Intriguingly, ERK1/2, but not the JNKs, was precipitously phosphorylated in microglial cells after IRT [36]. Also, ERK1 and ERK2 are directly interrelated with c‐JUN in vitro, and their phosphorylated forms were endogenously linked to c‐JUN in cells after IRT (Figure 1) [36]. Thus, both ERK1 and ERK2 are obligatory for IRT‐induced c‐Jun phosphorylation on Ser63 as well as Ser73 [36].

Furthermore, ERK 1 and ERK2 are autophosphorylable on both threonine as well as tyrosine residues, and the autophosphorylation associates with their stimulations [44]. Moreover, the stimulation of ERK1/2 via dual tyrosine as well as threonine phosphorylation by mitogen‐activated protein (MAP)/ERK kinase (MEK)1/2 resulted in the phosphorylation/activation of over 160 substrates following IRT [45]. However, autophosphorylation did not play a significant role in their IRT‐induced stimulation, due to their dependence on MEK1 function [36].

It is worth noting that MEK1 was stimulated in response to IRT, and MEK1 knockdown resulted in decreased phosphorylation of both ERKs as well as c‐Jun in IRT BV2 cells (microglial cells derived from C57/BL6 murine) [36]. Thus, IRT‐induced c‐JUN phosphorylation is determined by the MEK/ERK signaling pathway. Also, the stimulation of the ERK/c‐JUN signaling pathway was fundamental for the IRT‐mediated stimulation of numerous pro‐inflammatory genes in microglial cells (Figure 1) [36].

Mechanistically, the c‐JUN functions as a signaling amplifier via a positive feedback loop to amplify inflammatory gene secretion and consequently trigger IRT‐induced neuroinflammation [36]. Thus, the MEK/ERK/c‐JUN signaling pathway is very critical in IRT‐induced microglial inflammation responses. Furthermore, elevated concentration of ROS following IRT likely triggers the stimulation of ERK signaling in microglial cells, which was capable of modulating inflammatory gene secretion via the regulation of c‐JUN transcriptional activity (Figure 1) [36].

IRT‐induced stimulation of ERK1/2 signaling triggered the suppression of apoptosis in IRT cell by augmenting the secretion of antiapoptotic proteins, precisely Bcl‐xl, as well as preventing the activity of pro‐apoptotic proteins (Figure 1) [46]. Also, IRT‐induced stimulation of ERK1/2 was capable of up‐modulating CREB, which modulates the transcription of several genes responsible for the maintenance of neurogenesis, plasticity, as well as memory consolidation following the stimulation of tropomyosin receptor kinase A (TrkA) (Figure 1) [47].

Interestingly, while a TrkA‑specific inhibitor was capable of blocking *Calotropis gigantea* following IRT, the stimulation of NGF/TrkA/ERK1/2 signaling *C. gigantea* facilitated the synaptogenic potential of hippocampus neurons (Figure 1) [48]. Furthermore, NGF/TrkA signaling was capable of stimulating multiple intracellular signaling pathways, such as the MAPK, PI3K‑AKT, as well as phospholipase C*γ* cascades following IRT (Figure 1) [49]. Furthermore, the MAP kinase was also stimulated via TGF‐*β* following IRT [50].

## 4. JAK/STAT Signaling

The signal transducer and activator of transcription (STAT) family of transcription factors are located in the cytoplasm and consist of seven proteins such as STAT1, STAT2, STAT3, STAT4, STAT‐5a, STAT‐5b, as well as STAT6, which are stimulated via phosphorylation of signaling pathways such as cytokines, growth factors, as well as nonreceptor tyrosine kinases [51, 52]. On the other hand, the Janus kinase (JAK) pathway is associated with cytokine attaching to its related receptor, resulting in receptor dimerization subsequent to docking of JAK as well as phosphorylation of the receptor’s cytoplasmic tail [51, 52].

Among the STAT family of transcription factors, phosphorylation of STAT3 is constitutively stimulated or over‐secreted in diverse brain tumors [53, 54]. Notably, integral stimulation of STAT3 was crucial for medulloblastoma tumorigenesis via the regulation of target gene secretions, which were capable of protecting them against apoptosis and enhancing cell proliferation [55, 56]. Also, the JAK/STAT signaling pathway was associated with IRT triggering the expression of exosomes by glioma cells with proteins (Figure 1) [57]. Moreover, abnormal ribophorin II (RP‐II) secretion facilitated MCL1 secretion via the induction of STAT3, resulting in radioresistance (Figure 1) [54].

Furthermore, the stimulation of STAT3 facilitated anti‐apoptosis genes to suppress cell apoptosis involving MCL1 (Figure 1) [58]. Metabotropic glutamate receptor subtype 1 (mGluR1) associated signaling in IRT‐induced differentiation was dependent on STAT3 [59]. Notably, the IRT‐induced neuronal differentiation was intermediated by the STAT3‐mGluR1 pathway in C17.2 cells (mouse‐derived multipotent neural stem cells [NSCs] isolated from the cerebellum). Moreover, IRT‐stimulated neuronal differentiation was modulated by mGluR1 in C17.2 cells [59].

Furthermore, the mRNA as well as protein secretion of mGluR1 were inhibited by STAT3 blockade, but not by p53 blockade. Additionally, STAT3 induction of neuronal mechanisms was mediated by PI3K‐p53 signaling in Neuro‐2a cells (Figure 1) [60]. Also, cross‐talk between PI3K and mGluR1 stimulated the prevention of neuronal apoptosis [61]. Remarkably, IRT was able to trigger modifications in neuronal differentiation in undifferentiated neural stem‐like cells via PI3K/STAT3/mGluR1 as well as PI3K‐p53 signaling (Figure 1) [59]. Thus, IRT‐induced modification in neuronal differentiation was very critical in brain dysfunction triggered by IRT.

Intriguingly, the blockade of mGluR1 signaling reduced IRT‐induced neurite outgrowth, neuronal marker such as *β*‐III tubulin, as well as neuronal function‐related synaptophysin such as synaptotagmin1 and GABAA receptor, whereas the blockade of other mGluRs was not effective [59]. Also, IRT‐induced neurite outgrowth and *β*‐III tubulin secretion were reduced following the blockade of STAT3 or p53 [59]. It was initially established that the stimulation of STAT1 and STAT3 triggered the secretions of *β*‐III tubulin, synaptophysin, as well as growth‐associated protein‐43 (GAP‐43) (Figure 1) [62].

It was observed that the differentiation of motor neurons was blocked by STAT3 in human NSCs [63]. Moreover, IRT triggered the upregulation of *β*‐III tubulin‐positive cells as well as the reduction of nestin‐positive cells, and these changes were inhibited via the blockade of STAT3 or mGluR1 in primary NSCs (Figure 1) [59]. It is worth noting that not much research has been conducted on the involvement of the STAT signaling pathway in brain IRT, most especially in the neonatal or juvenile models. Thus, further research is warranted in this direction.

## 5. PI3K‐PKB/AKT Signaling

PI3K action is induced by various transforming genes as well as growth factor receptors, and its upregulation is considered in many tumors. On the other hand, AKT is a critical pro‐survival factor that inhibits the apoptotic signaling pathway as well as triggers pro‐survival pathways leading to cell development [64]. Also, AKT is first stimulated via the phosphorylation of Thr 308 at its kinase domain by the induction of 3‐phosphoinositide‐dependent protein kinase (PDK)‐1 and then via the phosphorylation of Ser 473 at its C‐terminal domain by PDK2 [46].

Thus, the PI3K/AKT signaling pathway is a ubiquitous as well as evolutionarily preserved signaling cascade that is associated with numerous cellular functions, such as cell proliferation, differentiation, migration, apoptosis, as well as metabolism [65, 66]. Furthermore, the stimulation of PI3K/AKT signaling is implicated in the poor prognosis in GBMs [67]. It is worth noting that the blockade of the PI3K signaling suppressed neurite outgrowth, secretion of *β*‐III tubulin as well as mGluR1, and the phosphorylation of AKT, p53, as well as STAT3, which are stimulated by IRT [59]. Thus, PI3K functions as an upregulator of STAT3/mGluR1/p53 in IRT‐stimulated neuronal differentiation (Figure 1) [59].

Noticeably, angiopoietin‐1 (ANG‐1) mitigated IRT‐stimulated neuronal apoptosis, whereas blockade of the PI3K/AKT/ANG‐1 pathway inhibited the protective effect of ANG‐1, signifying the pro‐survival function of the PI3K/AKT pathway following neuronal IRT (Figure 1) [14]. Moreover, miR‐711 augmentation triggered apoptosis via downstream targets in the pro‐survival PI3K/AKT pathway such as AKT1 and ANG‐1 (Figure 1) [68]. Notably, ANG‐1 exerted an anti‐apoptotic influence via the stimulation of AKT [68]. Interestingly, IRT stimulated upregulation of neuronal pro‐apoptotic miR‐711 with associated reductions in AKT as well as ANG‐1 [69].

Distinctively, simultaneous augmented concentrations of miR‐711, AKT1, as well as ANG‐1 were detected in the RISC, indicating that miR‐711 upregulation depended on the IRT‐stimulated downregulation of AKT as well as ANG‐1 [14]. Also, TrkA was capable of stimulating the phosphorylation AKT, which was considered as one of the crucial factors in the modulation of cell survival as well as apoptosis (Figure 1) [70]. Furthermore, the epidermal growth factor receptor (EGFR) inhibitor was capable of blocking IRT‐induced stimulation of AKT, which was determined by serum factors [65].

It is worth noting that the blockade of AKT, PI3K, as well as EGFR stimulation during IRT augmented the radiosensitivity of U87MG cells [65]. Also, phosphorylated EGFR correlated well with phosphorylated AKT, which supports the fact that EGFR is the key modulator for the stimulation of the PI3K/AKT pathway (Figure 1) [71]. Markedly, IRT induced the stimulation of EGFR as well as its downstream PI3K/AKT pathway similar to ligand activations [65]. Furthermore, response to IRT is influenced by the PI3K/AKT ability to modulate cell growth, proliferation, as well as survival [72, 73].

The radioresistance capabilities of PI3K/AKT activity in brain cancers in vitro, independent of TP53 status, as well as in vivo, is well established [65, 73]. Moreover, IRT is capable of triggering apoptosis via induction of hypoxia, and AKT is able to negatively modulate this process [74]. Notably, glycogen synthase kinase 3 (GSK3) is capable of stimulating hypoxia‐induced apoptosis via the stimulation of the mitochondria‐dependent death‐signaling pathway [75]. Intriguingly, AKT is able to block GSK3 via the blockade of phosphorylation at Ser 9, which triggers the induction of glycolysis as well as glucose transport that blocks hypoxia‐induced apoptosis (Figure 1) [76, 77].

## 6. p53 Signaling

The p53 is a critical transcriptional as well as nontranscriptional factor within a complex network that modulates cellular behavior as well as several biological activities [78]. Remarkably, p53 is frequently mutated in cancer although it is widely regarded as a tumor suppressor [79]. Also, it has been implicated to be beneficial in cell functions following stress response, such as cell‐cycle arrest, senescence, as well as apoptosis [78]. It was observed that IRT augmented gene secretion in the p53 signaling pathway via neuronal apoptosis in the pituitary (Figure 1) [80].

The p53 protein is up‐modulated in the central nervous system (CNS) after IRT‐stimulated DNA damage and played a critical role in speeding up apoptosis [81]. Thus, apoptosis was primarily regulated via a p53‐dependent apoptotic pathway in the developing brain, and radioresistance augmented appreciably during development due to the reduction in p53 mRNA secretion [82]. Also, the number of p53‐positive cells after IRT was much elevated in P9 compared to P23 animals, implying that the more immature the cells, the greater the dependency on p53 for cell death [83].

It was also established that p53 knockout salvaged the brain from IRT‐induced volume losses in the short term. However, normal growth in the p53 knockout‐IRT mice was hindered so much that the impairments persisted into early adulthood [84]. Also, it was further observed that none of the structures affected by IRT showed completely normalized outcomes as a result of p53 knockout with the exception of the DG [84]. Moreover, IRT‐dependent induction of neuronal p53 pro‐apoptotic pathways is supported by two mechanisms such as p53 phosphorylation/activation as well as blockade of pro‐survival AKT pathways [14].

The pro‐survival molecule AKT suppresses transcription of Bcl‐2 interacting mediator (BIM) of cell death and p53‐upregulated modulator of apoptosis (PUMA) and may counterbalance p53‐mediated apoptosis [85]. PUMA is a pro‐apoptotic protein that stimulates apoptosis via Bax as well as the mitochondria‐dependent pathway. IRT was capable of stimulating upregulation of pro‐apoptotic Bcl2 proteins subsequent to mitochondrial outer membrane permeabilization (MOMP) with cytosolic expression of cytochrome C as well as allograft inflammatory factor 1 (AIF‐1) also known as ionized calcium‐binding adapter molecule 1 (IBA1), thus triggering the intrinsic apoptosis pathway (Figure 1) [14].

Furthermore, 53BP1 in association with phosphorylated ATM as well as gH2AX constituted the formation of foci DNA damage marker proteins, which were detected in a 3D tissue model after IRT [86]. Markedly, foci diameter growth correlated with chromatin remodeling in facilitating DNA repair [86]. Hippo pathway‐related gene secretion in the pituitary gland was reduced appreciably after brain IRT. Interestingly, stimulation of the p53 pathway and blockade of the Hippo pathway following IRT triggered more apoptosis and reduced cell proliferation, eventually resulting in pituitary injury (Figure 1) [80].

## 7. Mammalian Target of Rapamycin (mTOR) Signaling

mTOR modulates cell proliferation, autophagy, as well as apoptosis via numerous signaling pathways in the body [87]. The mTOR kinase via the stimulation of AKT as well as MAPK blocks autophagy induction, whereas its blockade via AMPK and p53 facilitates autophagy induction [88]. Also, the mTOR/PI3K/AKT pathway is an intracellular signaling pathway that modulates cell cycle and may be crucial following cranial IRT (Figure 1) [89]. Furthermore, mTOR blockade decreased oxygen consumption via the blockade of the mitochondrial respiratory complex I [90].

Everolimus, an mTOR inhibitor, augmented IRT damage of tumor vasculature in an in vivo mouse tumor model but no in vitro radiosensitization of tumor cells grown in cell cultures [91–93]. Thus, mTOR inhibitors exert augmented IRT response by blocking angiogenesis [93]. It was further established that mTOR inhibitors augmented IRT response of tumor cells both in vitro and in vivo, irrespective of the timing as well as the schedule of mTOR blockade [94].

It is worth noting that blockade of mTOR decreased eukaryotic translation initiation factor 4E (eIF4E), a key factor in the translation that stimulates the configuration of the eIF4F cap complex obligatory for cap‐dependent translation [95]. Also, elevated eIF4E correlated with radioresistance, and selective silencing of eIF4E augmented radiosensitivity of cancer cell lines but not normal cells (Figure 1) [96]. Studies involving the IRT and mTOR signaling pathway in the CNS are very limited. Thus, more in vitro as well as in vivo studies on the affected cranial IRT on the mTOR signaling pathway are needed, especially in the neonatal or juvenile models.

## 8. NF‐κB Signaling

The NF‐*κ*B was initially recognized as a protein anchored to a sequence in the immunoglobulin *κ* light chain enhancer in B cells, and it is capable of stimulating numerous genes associated with stress responses, inflammation, as well as apoptosis [97]. Also, it is a heterodimer made up of p50 and RelA, which is a transcription factor associated with the modulation of inflammatory response following IRT [98, 99]. Furthermore, NF‐*κ*B is typically blocked by inhibitory *κ*B protein (I*κ*B), which sequesters it in the cytoplasm (Figure 1) [100].

Moreover, activated I*κ*Ks phosphorylate I*κ*B following stimulation, leading to its mortification facilitated by beta‐transducin repeat‐containing protein (*β*TrCP) (Figure 1) [100]. Thus, triggering the release of the sequestered NF‐*κ*B, which then translocated into the nuclei to stimulate the secretion of its target genes that facilitate survival as well as proliferation [100]. Also, IRT stimulates NF‐*κ*B to secrete cyclin D1, a cell cycle–specific genes implicated in radioresistance [99]. Moreover, NF‐*κ*B has specific DNA binding sequences that trigger cellular responses to IRT [101].

Furthermore, NF‐*κ*B regulates radioresistance via the modulation of IRT‐induced DNA DSBs repair as well as cell cycle arrest (Figure 1) [102]. Additionally, nuclear translocation of the NF‐*κ*B p65 subunit was associated with the upregulation of phosphorylated *γ*‐H2AX, which facilitates DSBs after IRT [103]. Besides, NF‐*κ*B essential modulator (NEMO) was noticeably elevated, while NF‐*κ*B regulatory inhibitor‐*α* (I*κ*B‐*α*) was substantially reduced after IRT. Moreover, stimulation of the NF‐*κ*B signaling pathway triggered microglial stimulation following IRT [103].

It is now established that IRT augments NF‐*κ*B activity, while NF‐*κ*B inhibitor augments IRT‐induced cell death [104]. Specifically, IRT profoundly activates NF‐*κ*B in human NB cells, resulting in induced radioprotection, and blockade of NF‐*κ*B augmented IRT‐induced cell death [105]. Thus, NF‐*κ*B in IRT cells determines radioresistance of the tumor cells [105]. It was further established that tumor cell proliferation, migration, as well as radioresistance were modulated by the NF‐*κ*B/HIF‐1 signaling pathway (Figure 1) [106]. Thus, blocking the activation of NF‐*κ*B is a potentially effective enhancing therapy for cranial IRT for blocking glioma growth [107].

Remarkably, mutual stimulation of NF‐*κ*B and tumor necrosis factor (TNF)‐*α* was obligatory for the inflammatory response induced by IRT [108]. Also, TNF‐*α* was capable of stimulating NF‐*κ*B and JNK, which requires TNF receptor–associated factors (TRAFs) that in turn interact with the downstream NF‐*κ*B‐inducing kinase (NIK) (Figure 1) [109, 110]. Furthermore, ERK was capable of stimulating NF‐*κ*B, triggering anti‐apoptosis, while the JNK and p38 pathways facilitated apoptosis (Figure 1) [108]. Interestingly, the ERK‐mediated anti‐apoptotic response was also dependent upon its cellular locations as well as interaction with NF‐*κ*B/I*κ*B complexes [111].

## 9. TGF‐*β* Signaling

TGF‐*β* is a multipurpose signaling that regulates developmental programs as well as modulates cell behavior in animal cells. The TGF‐*β*‑related cytokines have several effects on cell proliferation, differentiation, tissue homeostasis, morphogenesis, and regeneration, as well as the numerous diseases that result from their malfunctions [112]. TGF‐*β* is expressed in response to the injury, and its concentrations are upregulated in the brain IRBI [50]. The upsurge in the TGF‐*β* levels triggers the accumulation of proteins, resulting in microvascular disintegration [50]. The TGF‐*β* signaling is linked to binding to serine/threonine kinase receptors such a TGF_RI, which is type I, and TGF_RII, which is type II [50].

TGF‐*β*1 has been implicated in both the blockade of epithelial cell proliferation as well as the development of tissue fibrosis [113]. Also, tissue expression of TGF‐*β*1 is associated with an elevated plasma level [113]. Thus, normal tissue injury is mediated by TGF‐*β*1 as well as its elevated circulatory level generated by the tumor [114]. Notably, IRT of the brain triggered endothelial cells to stimulate apoptosis in NSCs via the TGF‐*β*/Smad3 signaling pathway in mouse models (Figure 1) [50]. Also, TGF‐*β* directly activates JAK/STAT3 in coordination via the Smad pathway. Moreover, TGF‐*β*1 is stimulated by IRT‐induced free radicals.

TGF‐*β*1 synthesis was augmented in brain stem–like endothelial cells on exposure to IRT and other injuries [50]. Also, elevated TGF‐*β*1 levels were associated with about a 60% decrease in neurogenesis in the hippocampus in mouse models [115]. Remarkably, IRT triggered overproduction of TGF‐*β*, which resulted in the loss of cognition due to the absence of neurogenesis in the hippocampal region of the brain [50]. Furthermore, TGF‐*β* downregulation via the stimulation of MAP kinase, JAK/STAT, and AKT pathways decreased neurogenesis, while stimulation of the ALK5 receptor–mediated pathway upregulated neurogenesis (Figure 1) [50, 116].

Moreover, TGF‐*β* as well as TNF‐*α* were capable of activating the MAPK signaling pathways following IRT (Figure 1) [117]. Interestingly, IRT was able to stimulate all three MAPK via TGF‐*β* in varying intensity [116]. Specifically, TGF‐*β* triggered MAP kinase stimulation via the ERK cascade pathway, such as ERK1 and ERK2, which are major subgroups of MAP kinase and are also known as MEKs and MKKs (Figure 1) [50, 116]. Additionally, the mechanism above triggers the stimulation of RAS, which in turn triggers activation of RAF (Figure 1) [116]. RAF1 in positive feedback then activates MAPK, which is associated with cell differentiation, proliferation, as well as apoptosis [116, 117]. Apoptosis resulted in deceased NSCs and, subsequently, impaired neurogenesis [117].

## 10. TNF Signaling

TNF is a multifunctional cytokine that regulates various cellular activities such as cell survival, proliferation, differentiation, as well as death. It has been implicated in inflammation‐associated carcinogenesis due to its pro‐inflammatory activities and its secretion by inflammatory cells. Also, TNF exerts its biological functions via the stimulation of distinct signaling pathways like NF‐*κ*B and JNK [118]. Markedly, IRT is capable of augmenting the secretion of the TNF receptor superfamily groups such as TNF‐*α* and p75 neurotrophin receptor (p75^NTR^) (Figure 1) [119–121].

TNF‐*α*, a member of the TNF receptor superfamily, serves as an intermediator of the immune system, safeguarding the host from the attacks of countless infections as well as tumor cells, and stimulates the death of tumor cells via apoptosis [122]. It was observed that IRT at low dose is able to trigger the production of TNF‐*α* in microglial cells as well as astrocytes in vitro studies [119]. Also, the production of TNF‐*α* was augmented in cultured monocytes obtained from patients who had undergone an IRT [123]. Furthermore, concomitant augmented secretion of TNF‐*α* and TGF‐*β*1 in the right cerebral hemispheres was observed following IRT, indicating that TNF‐*α* is a potent trigger of NF‐*κ*B activation by IRT [10]. Additionally, ERK activated by TNF‐*α* regulates NF‐*κ*B activation through I*κ*B kinase phosphorylation [124].

P75^NTR^, is also a member of the TNF superfamily, which is widely expressed in the developing CNS), and it is associated with neuronal survival, neurite outgrowth, as well as synaptic plasticity [125]. Also, p75^NTR^ has been implicated in the regulation of several intracellular pathways such as MAPKs, JNK, AKT, NF‐*κ*B, RhoA, PKA, as well as HIF (Figure 1) [126]. It was observed that p75^NTR^ mediates IRT‐induced learning as well as memory dysfunctions, suggesting that it is a negative regulator of hippocampal function [127]. Also, p75^NTR^ immunoreactivity revealed its co‐secretion with NeuN after pre‐ and post‐IRT exposure, with no connection between GFAP and p75^NTR^ in the hippocampus, signifying that p75^NTR^ is primarily located in hippocampal neurons (Figure 1) [121]. Furthermore, TAp73 was able to stimulate the augmentation of p75^NTR^ mRNA following IRT exposure, signifying that the TAp73/p75^NTR^ axis is critical in the pathogenesis of IRT‐induced cognitive dysfunction (Figure 1) [121].

## 11. ROS

The most critical component of IRT antitumor cell effects is the generation of ROS as well as nitrogen molecular species [9, 128]. Oxidative as well as nitrosative bursts are implicated in the restructuring of irreversibly cellular machineries like nucleic acids, proteins, as well as membranes that influence the redox state within cells via the modification of the ratio of oxidizing to reducing equivalents. The key pathways influenced by ROS are associated with redox signaling [128]. Also, electron transfer–associated cellular processes are stimulated via molecular dioxygen‐originating ROS produced through reactions like superoxide anion (O2^**•-**^), hydroxyl radical (HO^•^), or hydrogen peroxide (H_2_O_2_) [128].

Interestingly, the same ROS that are cytotoxic via redox‐sensitive moieties like thiol groups, which may modify protein structures at high concentrations and maintain cellular homeostasis at lower concentrations, and the process is regulated via complex antioxidant mechanisms [129]. ROS generation is one of the initial cellular responses after IRT [130]. It is worth noting that microglia are stimulated, which triggers immune cells to infiltrate the brain following IRT, and this process is depicted with ROS production as well as detoxification of normal physiological activities (Figure 1) [4, 131].

Also, IRT triggers the generation of ROS via oxidation of cellular water, leakage of the mitochondrial electron chain, or augmented stimulation of nicotinamide adenine dinucleotide phosphate oxidase (NADPH) oxidases (Figure 1) [3, 5, 36]. However, an imbalance between ROS generation as well as ROS elimination results in oxidative stress (Figure 1) [132, 133]. Several machineries of ROS trigger damage to key cellular components, like lipids, proteins, and DNA, resulting in cell death through necrosis or apoptosis (Figure 1) [134]. Thus, ROS contributes to neuronal toxicity as well as RIBI.

Furthermore, DNA damage and inflammation are the resultant effects of augmented ROS and free radicals like O_2_^•-^, HO^•^, or H_2_O_2_ (Figure 1) [4]. Notably, H_2_O_2_ induced oxidative stress as well as apoptosis in HT22 cells, which was associated with upregulated secretion of p‐ERK 1/2, p‐JNK, as well as p‐P38 [132]. Moreover, ROS were capable of stimulating the MAPK pathway as well as the NF‐kB pathway, which are fundamental for cell proliferation following IRT (Figure 1) [4, 50]. The stimulated MAPK signaling results in apoptosis, which triggers oxidative stress initiated by IRT [135].

It is also worth noting that enzyme stimulation, cycle modulation, as well as apoptosis are influenced by augmented Ca^2+^ concentration, which is capable of stimulating mitochondrial ROS formation (Figure 1) [136, 137]. Overall, ROS production is a critical factor associated with IRT‐induced acute as well as chronic brain injury [138]. Further studies on mitochondrial dysfunction and ROS in excitotoxicity as well as apoptosis significance in the pathogenesis of RIBI in the neonate and juvenile models are warranted.

## 12. Cluster of Differential Markers

**Figure 2:**
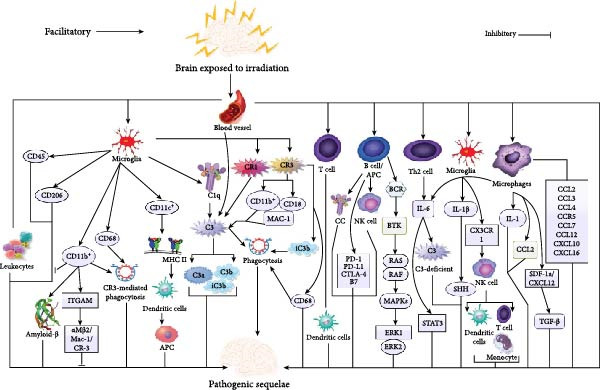
An illustration showing the key immune mechanisms associated with IRT‐induced neurological deficits or sequelae following brain IRT. *Note:* Refer to the text for detailed explanations. Also, refer to the general abbreviation list for the meaning of abbreviations.

Cluster of differentiation (CD) is a surface marker that recognizes a specific differentiation lineage identified by a cluster of monoclonal antibodies. CD antigens were molecules initially outlined as being present on the cell surface of leucocytes and identified by particular antibody molecules. They currently comprise intracellular molecules as well as molecules present on cells other than leucocytes. Notably, there were no variations in other phenotypic markers of microglia/macrophage stimulation such as CD68 and CD45, following IRT (Figure 2) [139].

Also, microglia isolated from IRT juvenile brains exhibited a more pro‐inflammatory profile than those from adult brains [140]. They exhibited decreased secretion of CD206, which signifies the presence of cell‐intrinsic properties pointing to the fact that the juvenile brain responds more robustly to an insult (Figure 2) [140]. Furthermore, CD68‐positive microglia were detected in the hippocampus 2 months after IRT to adult rat brains [133]. However, a substantial reduction in gene secretory levels of CD11b^+^ in the hippocampus of IRT mice was observed 7 days after IRT (Figure 2) [141, 142].

It was observed that microglia downregulate CD11b, resulting in efficient phagocytosis and clearance of *β*‐amyloid in an Alzheimer’s disease (AD) mouse model (Figure 2) [143]. Also, integrin alpha M (ITGAM), a protein subunit that forms the heterodimeric integrin alpha‐M beta‐2 (*α*M*β*2) molecule, also known as macrophage‐1 antigen (Mac‐1) or complement receptor (CR)‐3 that encodes CD11b, a marker of microglia stimulation, was downregulated following IRT (Figure 2) [141]. Moreover, CD11c^+^ cells exhibited positive staining for major histocompatibility complex (MHC)‐II, signifying that these cells were mature dendritic cells capable of functioning as antigen‐presenting cells (APCs) (Figure 2) [142].

Moreover, a well‐defined influence of fractionated IRT on the population of resting microglia secreting CD11b antigen was observed [141]. Interestingly, IRT resulted in a significant regression or deficiency of microglia until 90 days after IRT, which correlated with an unexpected regression or deficiency of stimulated microglia secreting CD68 antigen [144]. Thus, the CD11b marker is co‐secreted in resting as well as stimulated forms of microglia [141]. Also, CD68 and CD11b were upregulated by IRT in Ty1^+^ male mice, suggesting their potential for augmented CR3‐mediated phagocytosis of opsonized material that was deficient in CR3 knockout mice (Figure 2) [145].

## 13. Complement System

The complement cascade (CC) has recently been implicated in synaptic elimination during brain development as well as neurodegenerative diseases although it was originally thought to only regulate pathogenic immune responses and phagocytosis. Also, microglia have been implicated in being associated with synaptic interaction, which is dependent on the CC [145, 146]. Microglia are resident macrophages of the CNS, which perform functions such as the recognition of tagged components and consequent phagocytosis as well as elimination [145].

The function above is accelerated via opsonization, which occurs when C1q binds its target, triggering a protease cascade and augmentation of downstream complement C3 as well as C3 fragments such as C3a, C3b, and iC3b (Figure 2) [147, 148]. Furthermore, microglia integrally secrete C1q as well as CR1 and, upon stimulation, upregulate CR3 (such as CR3: CD11b/CD18; MAC‐1) and are capable of shedding C3 protein throughout the CNS (Figure 2). Moreover, microglia via CR3 identifies iC3b, internalizes the target, as well as eliminates it through phagocytosis (Figure 2) [147, 148].

Additionally, C1q and C3 are confined to synapses, accelerating microglia CR3‐associated phagocytosis as well as trimming during development (Figure 2) [147, 149]. Markedly, C3 deficiency resulted in augmented hippocampal‐dependent learning, signifying that C3‐dependent phagocytosis impedes learning as well as memory in young and aging mice [150]. Interestingly, genetic ablation of C3 inhibited stimulation of the complement system through any of the three stimulation pathways [151]. Pre‐IRT microglia depletion prevented cognitive decline as well as dendritic spine loss in the hippocampus [152].

Also, C3 deficiency correlated with elevated levels of pro‐inflammatory cytokines during the acute phase, elevated quantities of microglia as well as a trend toward augmented cell proliferation in the granule cell layer during the subacute phase, and an enhanced learning ability after IRT [153]. Furthermore, acute cell death in the neurogenic region correlated with a transient elevation of pro‐inflammatory chemokines as well as cytokines in the young rat brain during the acute response to IRT [154, 155].

It was established that CR3 knockout mice exhibited augmented levels of CD68 immunoreactivity following IRT, signifying these microglia had an augmented aptitude for phagocytosis, but they are unable to do so via CR3‐facilitated elimination (Figure 2) [145]. Thus, microglial CR3‐dependent phagocytosis of spines is very crucial during cranial IRT [145]. Furthermore, an upregulation of C3, as well as its accumulation along blood vessels, was observed following IRT [156]. Further studies on the role of CC and cranial IRT in the neonates of juveniles are needed since these categories of patients are more prone to learning and memory impedes following cranial IRT.

## 14. T Cells

T cells secret a receptor that has the capability of identifying different antigens from pathogens, tumors, as well as the environment and also conserve immunological memory as well as self‐tolerance. T cells are also implicated as key regulators of several inflammatory as well as autoimmune diseases. The peripheral immune system does not offer immunological protection to the CNS, and hence, it has no “immunologically privileged,” as described earlier.

However, it is well established that leukocytes infiltrate the normal CNS, and they have been implicated in the immunological surveillance of the parenchyma (Figure 2) [157]. Cranial IRT induced the recruitment of T cells in the C57BL/6 mouse brain (Figure 2) [142]. It was observed that T cells were capable of infiltrating the brain in response to IRT, but these were associated with perivascular cuffing as well as IRT necrosis (Figure 2) [158]. Also, a delayed augmentation in CNS T‐cell as well as dendritic cell populations was observed after cranial IRT [142]. Thus, IRT augmented T‐cell surveillance in the CNS and persisted for months post‐IRT [142].

Furthermore, both T cells as well as dendritic cells exhibited an inclination to the white matter [142]. Besides, T cells also exhibited contiguity to dendritic cells in white matter, signifying that they may be interrelating with one another (Figure 2) [142]. The contiguity of T cells and dendritic cells in white matter was observed in C57BL/6 mice as part of normal aging [159]. Further studies on the functions of specific subtypes of T cells and dendritic cells in neonatal or juvenile brains following cranial IRT are warranted.

## 15. B Cells

B cells are specialized APCs capable of producing antibodies that can detachedly modify the function of cancer cells, stimulate the CC, as well as facilitate natural killer cell (NKC)–associated tumor killing through antibody‐dependent cell‐mediated cytotoxicity (Figure 2) [160]. Also, B cells form a major section of the adaptive immune system, and programmed cell death protein (PD)‐1, PD‐L1, T‐lymphocyte‐associated protein 4 (CTLA‐4), and the B7 molecules are secreted on B cells (Figure 2) [160, 161].

Furthermore, B cell development is a chronological process comprising the assembly, secretion, as well as signaling of the B cell antigen receptor (BCR) (Figure 2). Markedly, IRT stimulated numerous pro‐survival signaling pathways associated with class‐switching, such as the BCR/Bruton’s tyrosine kinase (BTK) pathway interrelating with RAS/RAF/MEK/ERK (Figure 2) [160]. Also, IRT was capable of stimulating robust B‐cell infiltrates, and focal stereotactic IRT was superior to large field conventional IRT during the stimulation of tumor immune cell infiltrates [160]. Moreover, it is established that intratumoral B cells are capable of regulating anti‐tumor immune responses [162].

Interestingly, the frequencies of pro‐/pre‐B cells as well as immature B cells were augmented as well as upregulation of phenotypic and activation markers after IRT of BM‐derived B cells [160]. Furthermore, pre‐pro B cells as well as early/late‐pro‐B cells, analogous to the earliest stages of mouse B‐lymphocyte development, were the most IRT‐sensitive B‐cell subpopulations. Intriguingly, pre‐B cells were the most resistant to IRT among early stages of B cell subsets [160]. Whole‐body IRT was associated with an upsurge in surface immunoglobulin on B cells in mouse models [163]. Further studies into the association of B cells and cranial IRT in neonatal and juvenile models are needed.

## 16. Interleukins (ILs)

ILs are a group of cytokines or signaling molecules originally thought to be solely expressed by white blood cells (WBCs) as a means of interaction. Currently, a wide variety of cells have been implicated in their production, and their functions are at and beyond diverse levels of the immune system [164]. It is worth noting that IL‐1, produced by macrophages, stimulates the acute phase reaction as well as fever, while IL‐6, produced by macrophages and type II helper T (Th2) cells, is a pleiotropic cytokine modulating several inflammatory as well as immunologic processes and stimulates the acute phase reactions to injuries (Figure 2) [113].

Notably, IL‐1*β* as well as IL‐6 were upregulated only in C3‐deficient brains following IRT (Figure 2) [145]. Interestingly, microglia isolated from IRT juvenile brains exhibited a more pro‐inflammatory profile than those from adult brains with more augmented IL‐1*β* secretion (Figure 2) [140]. Also, IL‐1*β* is able to increase BBB permeability by inhibiting astrocytic sonic hedgehog (SHH) production as well as obliterating the protective influence of astrocytes on BBB integrity [165]. Furthermore, augmented IL‐1*β* adversely influenced hippocampal neurogenesis by forming an inflammatory microenvironment that skews neurogenesis toward gliogenesis (Figure 2) [166].

Additionally, IL‐1*β* is directly interrelated with neural progenitor cells to trigger cell cycle arrest [167]. It is worth noting that IL‐6 has been implicated in brain development because its levels were augmented during normal brain growth [168]. Also, a decrease in IL‐6 was detected after IRT to the juvenile but not the adult rat brain, indicating that the young as well as adult brain respond contrarily to IRT [168]. Thus, cranial IRT in neonatal or juveniles triggered depletion of IL‐6, which is detrimental to normal brain cells.

Remarkably, IL‐6 modulates glioma characteristics like blockade of sequelae like apoptosis, facilitation of survival, proliferation, angiogenesis, invasiveness, as well as metastasis (Figure 2) [169]. It is also identified as a modulator of glioma metabolism. It is established that IL‐6 signals facilitate STAT3 stimulation in GBM cells in vitro, and targeting of either STAT3 or IL‐6 reduces GBM cell survival (Figure 2) [170]. Also, IL‐6 signals were associated with the stimulation of STAT3, which was also crucial for glioma stem cells’ survival (Figure 2) [169].

## 17. Chemokines

Chemokines are an immense group of small, expressed proteins that signal via cell surface G protein–coupled heptahelical chemokine receptors [171]. They are also referred to as chemotactic cytokines. The subfamily description includes CC, CXC, CX3C, or XC, followed by the letter L, which symbolizes “ligand,” and then a number according to when the gene was first discovered [172]. They are capable of inducing the migration of cells, such as leukocytes, which are components of WBCs.

There is currently evidence that IRT induced infiltration of bone marrow–derived cells into the hypothalamus by means of chemokine axes in the brain and bone marrow [173]. Also, they have been implicated in the development as well as homeostasis of the immune system and are associated with the protective or destructive immune as well as inflammatory responses [171]. Monocytes are critical facilitators of innate immune function because of their capacity to differentiate into tissue macrophages [174].

They are categorized into two distinct sub‐populations due to their secretion of specific cell surface antigens. Specifically, they grouped into “inflammatory” such as Ly‐6C^hi^CCR2^+^CX3CR12 as well as “circulating” such as Ly‐6C^lu^CCR22CX3CR1^+^ monocytes [175]. The inflammatory monocytes, which are capable of secreting C–C chemokine receptor type 2 (CCR2), are able to migrate from bone marrow, infiltrate damaged tissues where they transform into macrophages, and generate extreme concentrations of pro‐inflammatory cytokines [174, 175].

CCR2, which is integrally secreted by cells of the monocyte–macrophage lineage, has been implicated in IRT‐induced cognitive impairments [139]. Also, a clear distinction between the innate immune response in the brain following cranial IRT such as CX3CR1^+^, which is resident, and CCR2^+^, which is peripheral, was observed in CCR2RFP^−/+^CX3CR1GFP^−/+^ in mice [176]. Furthermore, IRT‐induced interruption in neuronal networks, accompanied by learning as well as memory and inflammatory response, was blocked in CCR2‐deficient mice following cranial IRT [176].

The CCR2‐deficient mice had decreased levels of IRT‐induced inflammatory response in the hippocampus, as well as augmented cognitive function as compared to wild‐type animals [139]. Thus, CCR2 is very critical in IRT‐induced hippocampal neuronal dysfunction, either directly or via the regulation of other pathways [176]. Notably, CX3CR1 is secreted solely by microglia in the CNS, whereas in the periphery, it is found on “resident” monocytes, NK cells, T cells, as well as dendritic cells (Figure 2) [140, 177]. However, CX3CR1 is not on “inflammatory” monocytes phenotypically recognized as Ly6C^hi^CCR2^+^CX3CR1 and normally identified in inflamed tissues [140, 178]. It was observed that the secretory levels of pro‐inflammatory genes, such as CXCL10, CXCL16, CCL2, CCL3, CCL4, CCL7, CCL12, and CCR5, were expressively augmented after IRT (Figure 2) [80].

Moreover, the protein secretion of CXCL10, CCL2, CCL7, CCL3, and CXCL2 was expressively augmented after IRT [80]. Interestingly, CCL2 was upregulated in both wild‐type and C3^−/−^ mice after IRT. Additionally, TGF‐b as well as stromal cell‐derived factor‐1/CXC chemokine ligand 12 (SDF‐1a/CXCL12) directed migration of adult hematopoietic stem and progenitor cells toward glioma cells in vitro and their homing to experimental gliomas in vivo [179] (Figure 2). Hypoxia is a crucial part of the gliomas milieu, and IRT is a crucial aspect of the typical therapy. Further studies into the association of chemotactic cytokines and cranial IRT in neonatal and juvenile models are needed.

## 18. Conclusion

IRT‐induced DSBs stimulate the interplay of key signaling pathways such as MAPK, JAK/STAT, PI3K‐PKB/AKT, p53, mTOR, NF‐kB, TGF‐*β*, TNF, as well as ROS to either trigger radiosensitization or radioresistance, as well as RIBI mechanisms. Also, IRT is capable of influencing fundamental immune players like CD markers, the CC, T cells, B cells, ILs, as well as chemotactic cytokines.

**Nomenclature**
53BP1: p53 binding protein 1
AD: Alzheimer’s disease
AIF‐1: Allograft inflammatory factor 1
ANG‐1: Angiopoietin‐1
AP‐1: Activator protein‐1
ATM: Ataxia‐telangiectasia mutated
BBB: Blood–brain barrier
Bcl‐2: B‐cell lymphoma 2
BCR: B‐cell antigen receptor
*β*TrCP: Beta‐transducin repeat‐containing protein
BIM: Bcl‐2 interacting mediator
BORA: Borealis
BTK: BCR/Bruton’s tyrosine kinase
CC: Complement cascade
CCR2: C–C chemokine receptor receptor type 2
CD: Cluster of differentiation
CNS: Central nervous system
CREB: cAMP response element‐binding protein
CR: Complement receptor
CXCL12: CXC chemokine ligand 12
DDR: DNA damage response
DDSs: DNA damage sensors
DSBs: Double‐strand breaks
EGFR: Epidermal growth factor receptor
eIF4E: Eukaryotic translation initiation factor 4E
ERK: Extracellular signal‐regulated kinase
GAP‐43: Growth‐associated protein‐43
GSK3: Glycogen synthase kinase 3
H_2_O_2_: Hydrogen peroxide
HO^•^: Hydroxyl radical
IBA1: Ionized calcium‐binding adapter molecule 1
I*κ*B: Inhibitory *κ*B protein
I*κ*B‐*α*: NF‐*κ*B regulatory inhibitor‐*α*
ILs: Interleukins
IRIF: IRT‐induced foci
IRT: Irradiation
ITGAM: Integrin alpha M
JAK: Janus kinase
JNKs: c‐Jun N‐terminal kinases
Mac‐1: Macrophage‐1 antigen
MAP: Mitogen‐activated protein
MAPK: Mitogen‐activated protein kinases
MDC1: Mediator of DNA damage checkpoint 1
MHC: Major histocompatibility complex
mGluR1: Metabotropic glutamate receptor subtype 1
MOMP: Mitochondrial outer membrane permeabilization
MRE11: Meiotic recombination 11
mTOR: Mammalian target of rapamycin
NADPH: Nicotinamide adenine dinucleotide phosphate oxidase
NEMO: NF‐*κ*B essential modulator
NF‐*κ*B: Nuclear factor kappa B
NHEJ: Nonhomologous end‐joining
NIK: NF‐*κ*B‐inducing kinase
NKC: Natural killer cell
NSCs: Neural stem cells
O2^•^‐: Superoxide anion
p53: Protein 53
PD: Programmed cell death protein
PDK‐1: 3‐phosphoinositide‐dependent protein kinase
PI3K: Phosphoinositide‐3‐kinase
PKB/AKT: Protein kinase
PUMA: p53‐upregulated modulator of apoptosis
RIBI: Radiation‐induced brain injury or damage
RISC: RNA‐induced silencing complex
ROS: Reactive oxygen species
RP‐II: Ribophorin II
SAPK: Stress‐activated protein kinase
SDF‐1a: Stromal cell‐derived factor‐1
SHH: Sonic hedgehog
SNF2: Sucrose nonfermenting 2
Sp‐1: Specificity protein 1
STAT: Signal transducer and activator of transcription
TGF‐*β*1: Transforming growth factor beta 1
Th2: Type II helper T
TNF: Tumor necrosis factor
TRAFs: TNF receptor–associated factors
TrkA: Tropomyosin receptor kinase A
UCHL3: Ubiquitin carboxyl‐terminal hydrolase isozyme L3
WBCs: White blood cells
*α*M*β*2: Alpha‐M beta‐2.

## Ethics Statement

The author has nothing to report.

## Consent

The author has nothing to report.

## Conflicts of Interest

The author declares no conflicts of interest.

## Author Contributions

**Seidu A. Richard:** study concepts and design, data acquisition, diagram preparation, manuscript preparation and editing.

## Funding

The author received no specific funding for this work.

## Data Availability

Data sharing is not applicable to this article as no datasets were generated or analyzed during the current study.
